# Mucinous adenocarcinoma derived from villous adenoma of the kidney with muconephrosis

**DOI:** 10.1002/ccr3.8397

**Published:** 2024-01-03

**Authors:** Tsubasa Kawamoto, Masanori Ishida, Takashi Yorozu, Elly Arizono, Yukari Wakabayashi, Toshitaka Nagao, Yoshio Ohno, Kazuhiro Saito

**Affiliations:** ^1^ Department of Radiology Tokyo Medical University Hospital Tokyo Japan; ^2^ Department of Pathology Tokyo Medical University Hospital Tokyo Japan; ^3^ Department of Urology Tokyo Medical University Hospital Tokyo Japan

**Keywords:** kidney, mucin, mucinous adenocarcinoma, pelvis, villous adenoma

## Abstract

Upper respiratory tract villous adenoma (VA) with muconephrosis is rare and should be included in the differential diagnosis when pelvic dilatation with a solid component is detected. VA may transform into malignant mucinous adenocarcinoma, which should be suspected if contrast enhancement on computed tomography (CT)/magnetic resonance imaging (MRI) and restricted diffusion on MRI are observed.

## Graphical abstract

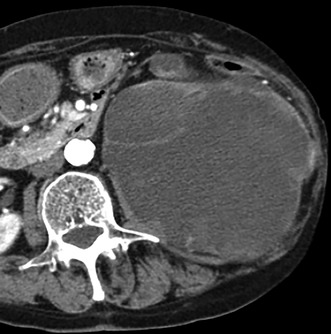

## 1 INTRODUCTION

Villous adenoma (VA) rarely occurs in the upper urinary tract.^1^, ^2^, ^3^, ^4^ The tumor is similar to VA in the large intestine which consists of goblet‐type mucin‐producing cells. VA in the upper urinary tract may be observed as muconephrosis in clinical practice^5^, ^6^, ^7^ and has the potential for malignant transformation because of the adenoma‐carcinoma sequence. Therefore, careful monitoring of this rare condition is mandatory.

However, because of its rarity, the detailed characteristics of VA of the renal pelvis and ureter and its malignant component have not been fully elucidated in the contemporary literature. To date, there are only 15 cases of VA of the renal pelvis reported in the (English) literature.^8^ Additionally, to the best of our knowledge, there is no detailed report with both multimodal images and genetic mutations of this pathologic entity.

Herein, we present the case of an 85‐year‐old woman who underwent surgery for a renal mass that was pathologically diagnosed as an adenocarcinoma derived from a VA and discuss the clinical and imaging features of VA and its malignant transformation.

## 2 CASE REPORT

A left renal mass was detected on abdominal ultrasonography during the first health examination in an 85‐year‐old Japanese woman. A medical interview revealed that she had sometimes experienced lower abdominal pain for a few months, and ultrasonography revealed left pelvis dilation. We recommended further examination to determine whether surgical resection was necessary. However, she wished to be under observation due to her advanced age. Four days before surgery, she developed severe left‐sided abdominal pain to the extent that she had difficulty standing and visited our hospital again. We considered the pain to be caused by the previously discovered left renal lesion and decided to remove the mass at her request. Various examinations were performed prior to the surgery. She had a medical history of chronic heart failure, hypertension, hyperlipidemia, hyperuricemia, and osteoporosis. Physical examination showed no abnormal physical findings other than tenderness in her left abdomen.

Preoperative laboratory blood examination revealed that her blood cell count, liver enzymes, blood coagulation function, and tumor markers (such as carbohydrate antigen 19–9 and carcinoembryonic antigen) were within normal limits. Blood urea nitrogen and creatinine were also within normal limits (11.0 mg/dL and 0.74 mg/dL, respectively). Her estimated glomerular filtration rate was slightly low (55.7 mL/min/1.73m^2^), and her C‐reactive protein level was slightly high (1.64 mg/L). Urinalysis revealed proteinuria and occult blood cell presence. Urinary cytology was negative for high‐grade urothelial carcinoma.

Abdominal ultrasonography revealed that the tumor of the left kidney had a homogenous low‐echogenicity tone with layered echoes and septa. We considered the mass to be a cystic‐like lesion.

**FIGURE 1:**
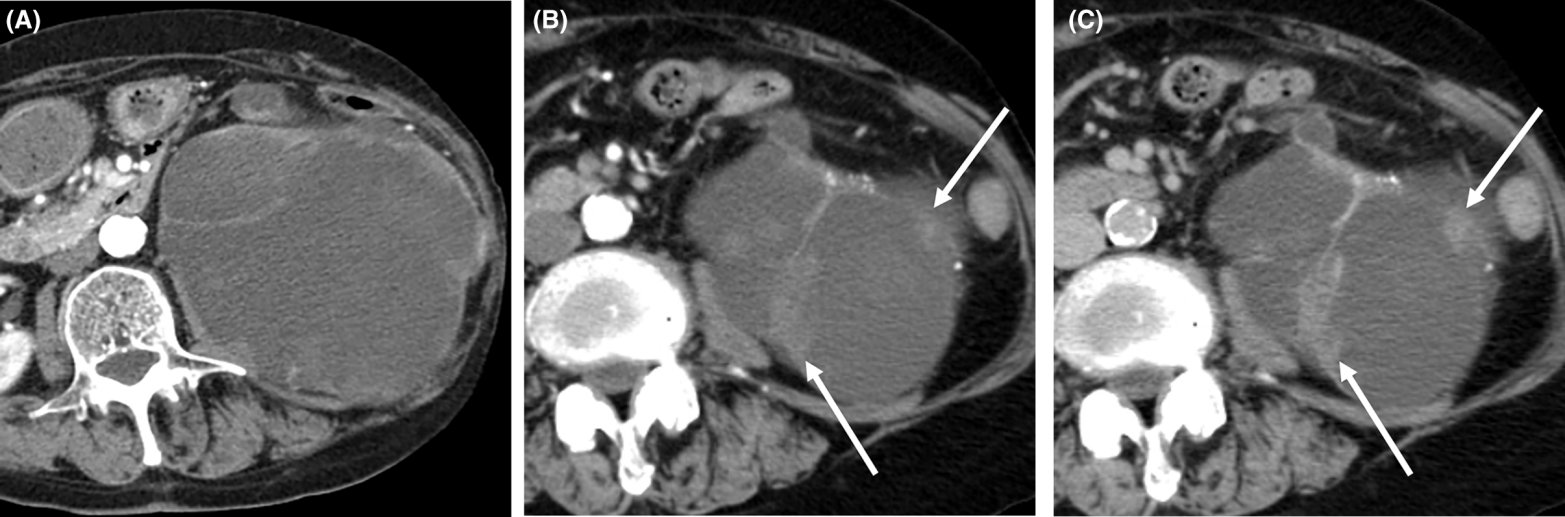
Contrast‐enhanced CT of the dilated renal pelvis and mural nodular lesions. The dilated renal pelvis is the main part of the lesion and renal parenchyma thinning (A). Irregular soft tissue parts with gradual enhancement at the wall of the dilated pelvis are shown arrows, (B) arterial phase; (C) delayed phase.

Abdominal dynamic contrast‐enhanced computed tomography (CT) was performed using a fixed‐injection‐period technique. The plain and contrast‐enhanced CT demonstrated a mass measuring 120 mm in diameter in the left kidney (Figure 1A). The low‐attenuation area, which was the majority of the mass, was approximately 20 Hounsfield units. The mass was accompanied by an irregular soft tissue density found mainly at the margins. An enhancement was observed in the wall and septa of the mass. There was also a gradual enhancement of the irregular soft tissue parts of the mass on dynamic contrast‐enhanced CT (Figure 1B,C). The enhancement of the solid portion was heterogeneous and without wash‐out. Thinning of the renal parenchyma and a ureteral duplication were also identified. The mass and one of the two ureters were contiguous, and this ureter was dilated. Therefore, the substance of the mass was considered to be the renal pelvis.

**FIGURE 2:**
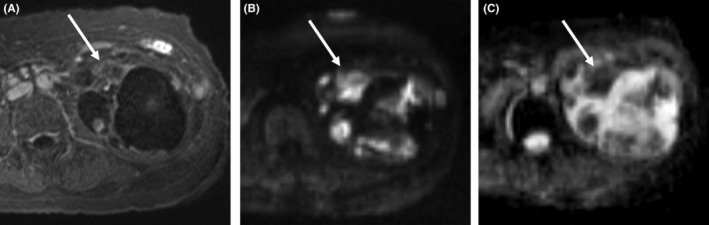
Contrast‐enhanced MRI on the arterial phase showing an irregular soft tissue part (arrows) with contrast enhancement (A) and restricted diffusion (B), diffusion‐weighted image (*b* = 800); (C) apparent diffusion coefficient (ADC) map, (ADC = 0.9–1.1 × 10^−3^ mm^2^/s).

On magnetic resonance imaging (MRI), the majority of the mass exhibited a low signal intensity on T1‐weighted images and a high signal intensity on T2‐weighted images; thus, it was considered a cystic lesion. A low signal intensity on T2‐weighted images located at the solid portion of the mass showed gradual enhancement on dynamic contrast‐enhanced MRI and diffusion restriction (apparent diffusion coefficient = 0.9–1.1 × 10^−3^ mm^2^/s) (Figure 2A–C).

**FIGURE 3:**
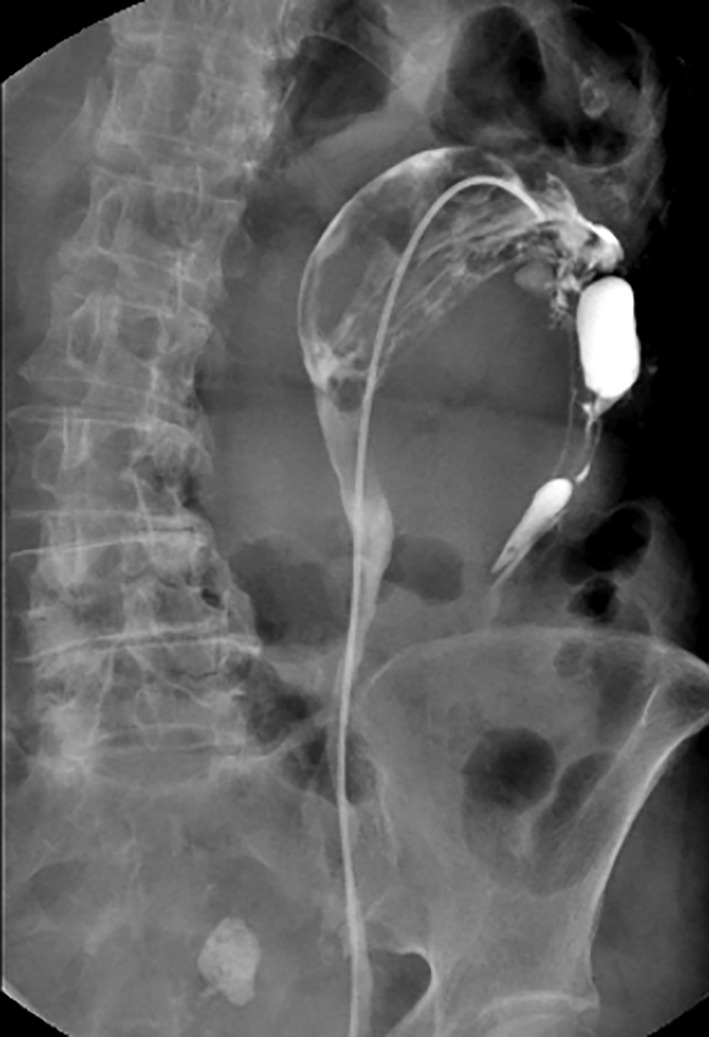
Retrograde pyelography showing the continuity between the ureter and the dilated pelvis forming the main part of the tumor.

Retrograde pyelography was performed, which revealed continuity between one of the ureters and the dilated pelvis forming the main part of the mass (Figure 3). At the time of this examination, discharge of mucinous urine from the ureteral orifice was observed by cystoscopy.

Based on imaging findings, the mass was considered to be the dilated pelvis and have a malignant tumor due to the irregular soft tissue component with enhancement on CT/MRI and diffusion restriction. The preoperative diagnosis was urothelial carcinoma of the renal pelvis or mucinous adenocarcinoma complicated with hydronephrosis or muconephrosis.

She subsequently underwent a left nephroureterectomy. The descending colon was compressed by the tumor. Since there was adhesion between the tumor and the descending colon as well as suspected tumor invasion, a partial colon resection was also performed. There were no intraoperative or postoperative complications.

**FIGURE 4:**
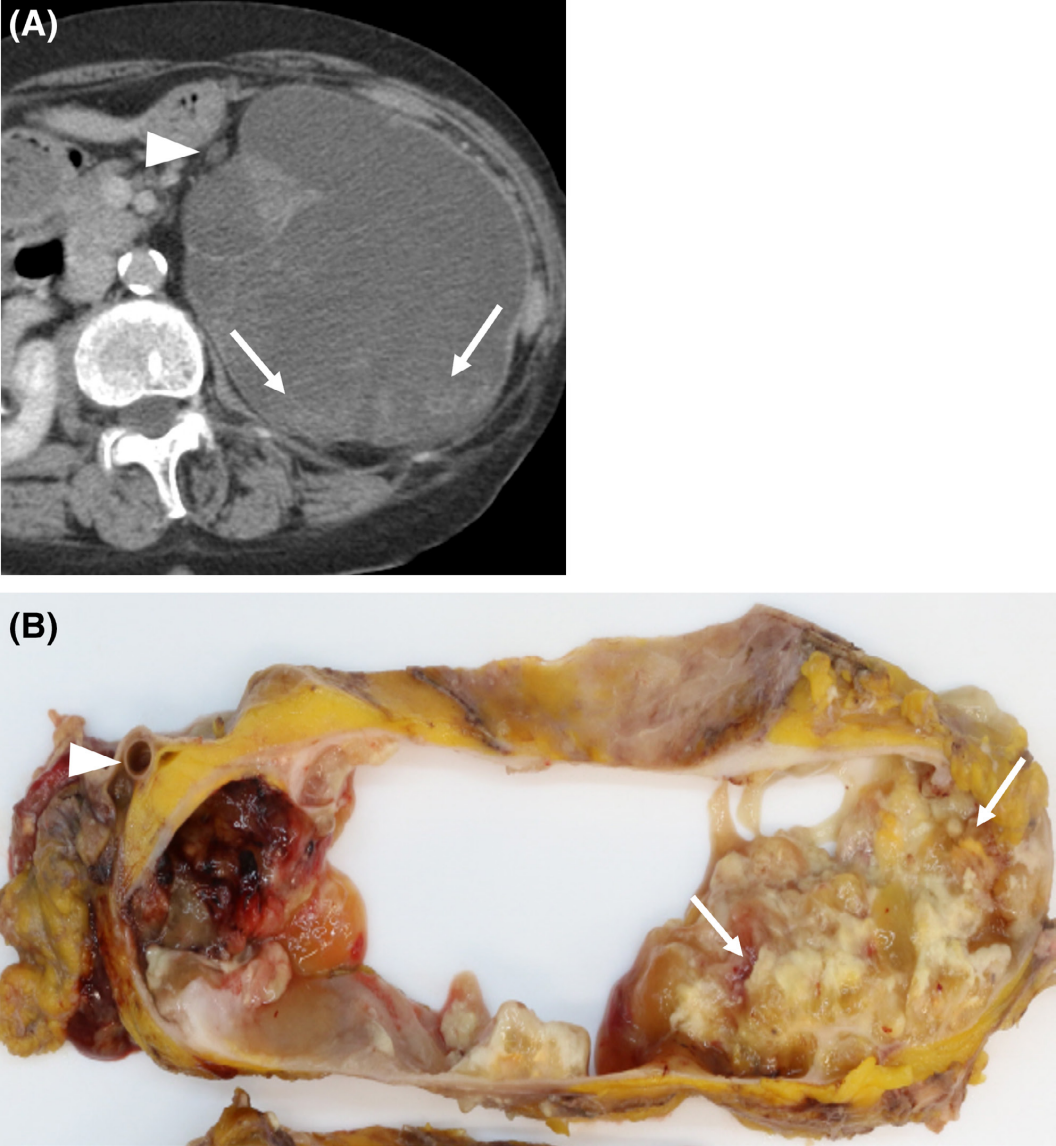
Exophytic papillary lesions along the wall of the dilated pelvis (arrows) compared between a CT image (A) and a macroscopic specimen (B); mucus has already been drained.

**FIGURE 5:**
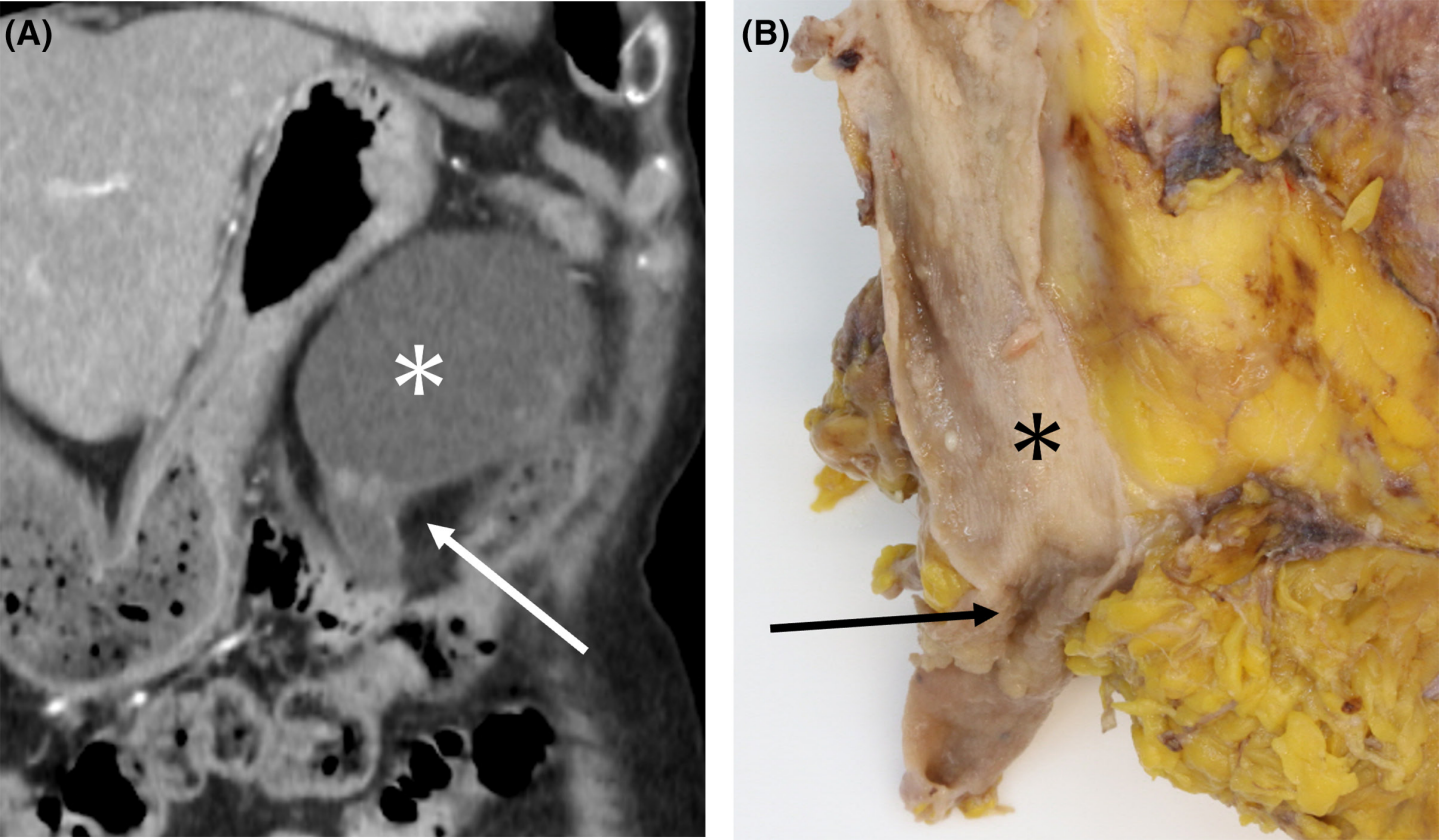
Correlation between CT and a gross pathological finding of stenosis of the pyeloureteral junction due to the presence of the tumor (arrows), (A) contrast‐enhanced CT image; (B) macroscopic image and the dilated renal pelvis (asterisks).

**FIGURE 6:**
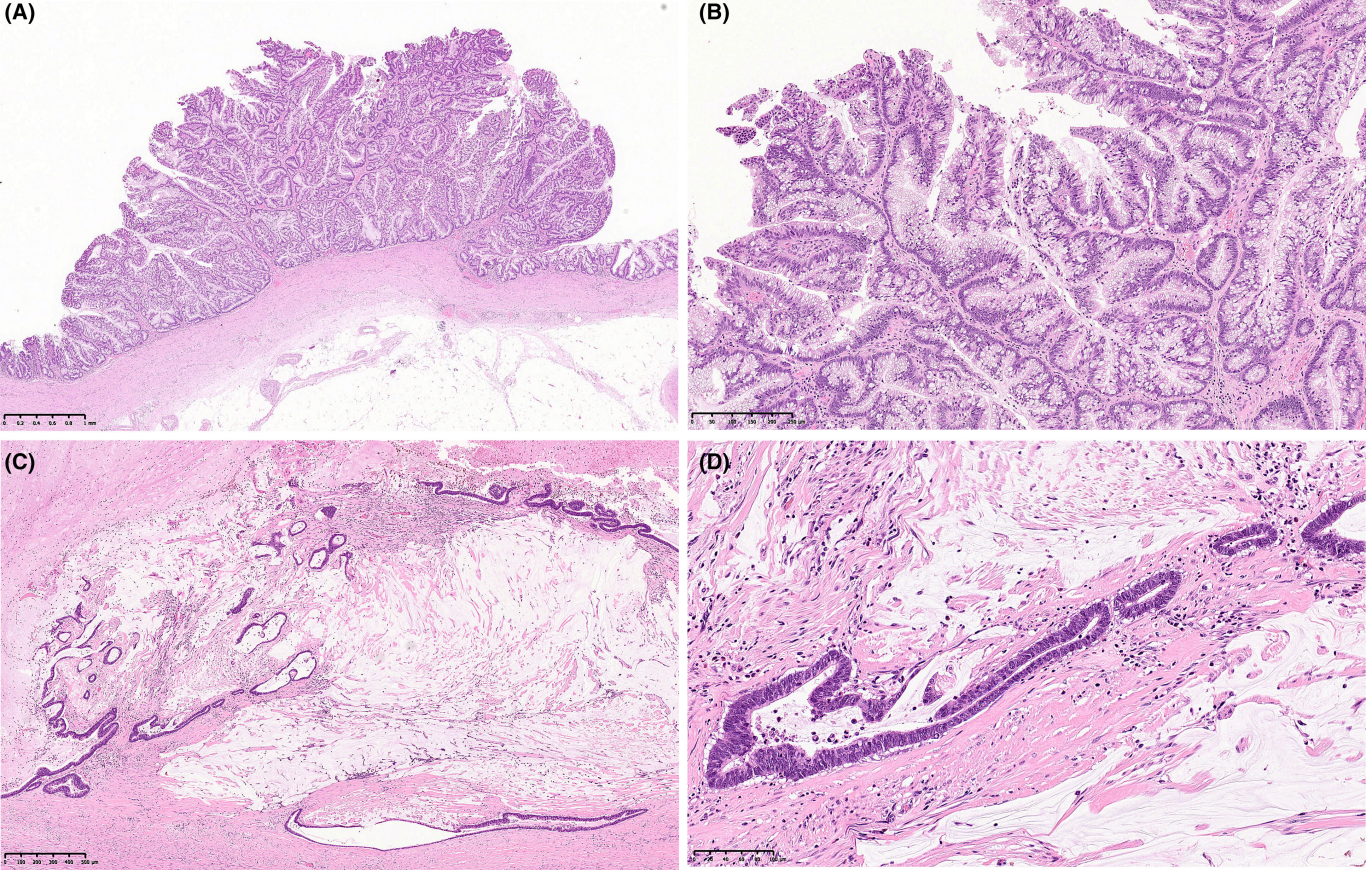
Histopathology of villous adenoma of the renal pelvis and pyeloureteral junction revealing typical villous structures. The villi replace the urothelium of the ureter without stromal invasion (A; hematoxylin and eosin [H&E], ×20). An adenomatous polyp composed of closely packed slender villi lined by columnar epithelium with intracellular mucin (B; H&E, ×100). Mucinous adenocarcinoma with abundant mucin (C; H&E, ×40). Mucinous adenocarcinoma with enlarged, hyperchromatic nuclei (D; H&E, ×200).

**FIGURE 7:**
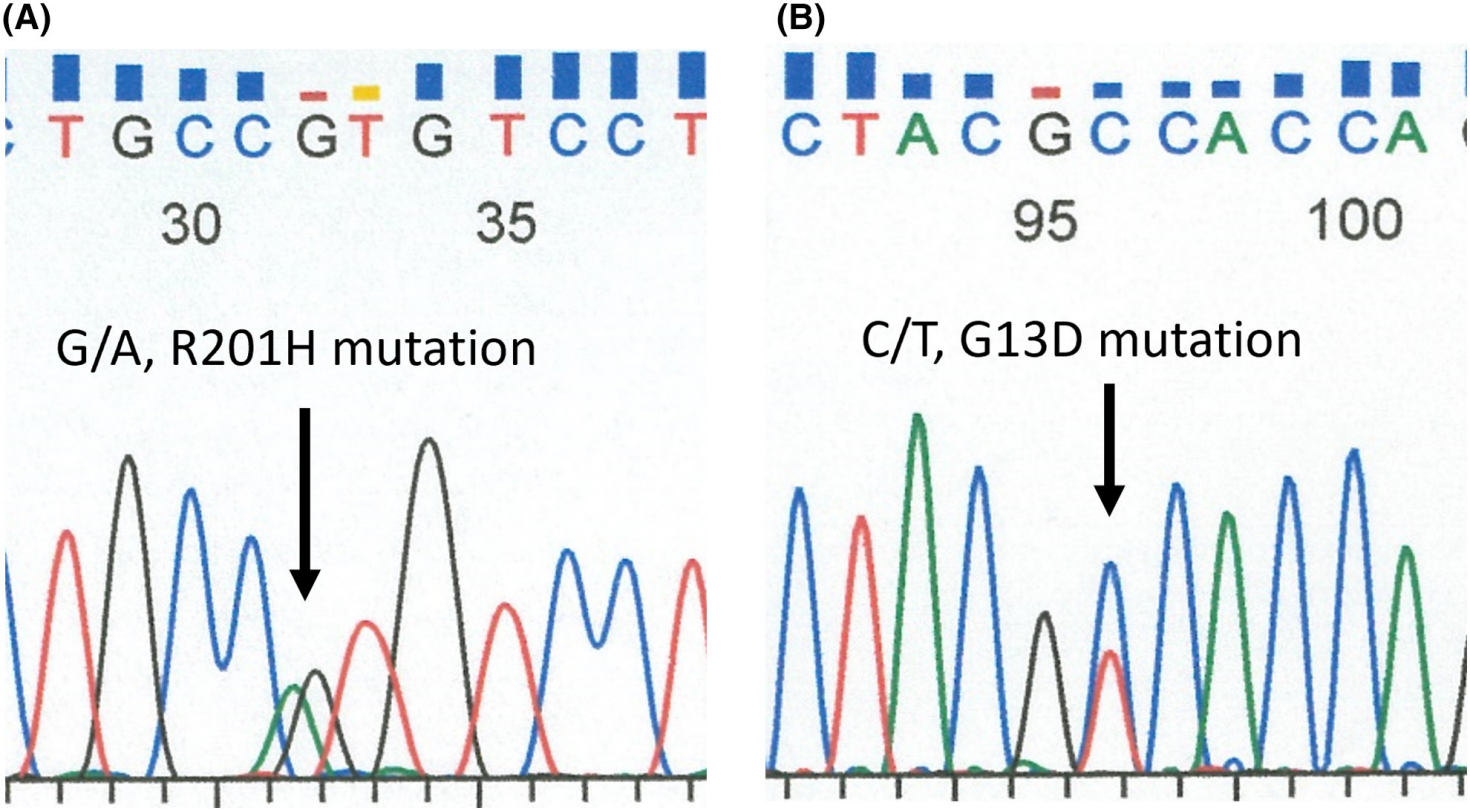
Sequence analyses of genetic mutations (A; GNAS [R201H] mutation [wild type; CGT], B; KRAS [G13D] mutation [wild type; GGC]) by the reverse‐transcription polymerase chain reaction method.

Gross pathological specimens demonstrated a mass measuring approximately 14 cm in diameter (Figures 4 and 5). The mass was formed in a markedly dilated pelvis and filled up with mucinous fluid. An irregular solid nodule was identified alongside the wall of the dilated pelvis (Figure 4B) and extended to the pyeloureteral junction (Figure 5B). The renal parenchyma was thinned out. The microscopic examination revealed that the surface of the mass was composed of villous components with irregular folding and exophytic growth (Figure 6A). Within the dilated renal pelvis and contiguous ureter, the tumor had villous structures lined by atypical columnar epithelium and many of the cells had intracellular mucin (Figure 6B). The epithelium showed enlarged, hyperchromatic nuclei, with varying degrees of nuclear spindling and stratification. Mixed in with this, the invasive component was observed focally in the form of mucinous adenocarcinoma with abundant extracellular mucin, dissecting the stroma of the renal pelvis wall (Figure 6C,D). Immunohistochemically, the tumor cells tested positive for MUC‐2 and MUC‐5AC, focally positive for CDX‐2, and negative for MUC‐1 and MUC‐6. MUC2 is found in the epithelium of the small and large intestines, MUC5AC is found in the epithelium of the stomach and colon, and CDX‐2 is found in the epithelium from the duodenum to the rectum. The fact that these tissues were positive for these cell types suggests that the tumor had formed a mucous layer. In addition, genetic analyses revealed GNAS and KRAS mutations of the tumor in the pelvis and urethra (Figure 7). These genetic mutations indicate a pathogenesis leading to invasive carcinoma.

The final diagnosis was mucinous adenocarcinoma derived from VA. The patient was able to return home 1 week after surgery without complications. At this point, there was no decrease in her renal function or appearance of anemia, and she was happy to be able to live her normal daily life, both of which indicated a positive outcome. However, 18 months postoperatively, a pulmonary nodule appeared and showed a tendency to enlarge, and it was considered a pulmonary metastasis. Subsequently, treatment for pulmonary metastasis was not performed according to the patient's wishes. Currently at postoperative 2 years, she is able to live alone and perform normal daily activities despite having pulmonary metastasis.

## 3 DISCUSSION

Although VA most commonly arises in the gastrointestinal tract, it can be found throughout the urinary tract in rare cases. VA of the urinary tract is histologically indistinguishable from its colorectal counterpart,^1^, ^2^, ^3^, ^9^ and chronic inflammation is considered one of the risk factors for the occurrence of VA.^1^, ^5^ In most cases, the patients have prototypical low‐grade dysplasia; however, associated high‐grade dysplasia, carcinoma in situ, and invasive adenocarcinoma may occur. A subset of VA in the urinary tract comprises precursor lesions that subsequently progress to adenocarcinoma and invasive adenocarcinoma.^10^, ^11^, ^12^, ^13^ Since there are no summarized reports on VA of the renal pelvis and ureter or this malignant transformation, one review article on villous tumors of the gastrointestinal tract can be informative.^14^ Ten cases of VA of the renal pelvis have been reported in English,^2^, ^4^, ^5^, ^8^, ^15^, ^16^, ^17^, ^18^, ^19^ only one of which had adenocarcinoma as a complication.^8^

In the present case, an irregular component within a large cystic lesion on CT and MRI was considered a solid component. Dynamic contrast‐enhanced CT and MRI showed gradual enhancement, suggesting the presence of much interstitial tissue. Although it was difficult to clearly distinguish between adenoma and adenocarcinoma on CT and MRI, the diffusion‐restricted areas on MRI reflected a high cell density, which may indicate carcinoma. With reference to the present case, it is clinically significant to recognize that VA can be detected on imaging when a dilated pelvis is detected, and it is essential to differentiate VA from adenocarcinoma; however, the identification of dysplasia or malignant foci is limited, as well as the presence of malignant foci. Therefore, the surgical resection of these lesions is ultimately necessary. Because of the potential for malignant transformation of VA, early resection is preferable when a dilated, mucus‐filled renal pelvis is detected, as in this case.

The instructive aspect of this case report is that the sizable pelvic dilatation is thought to be due to the combination of the abundant mucin produced by the VA located at the pyeloureteral junction, resulting in ureteral stenosis. It is important to exclude urothelial carcinoma to diagnose mucinous adenocarcinoma of VA origin. It is not uncommon for urothelial carcinomas to differentiate into glandular structures which also produce mucin. However, abundant mucinous production is unlikely to result in sizable pelvic dilatation as in the present case. In addition, no urothelial carcinoma component was found on histopathology. From this perspective, in the present case, it is more likely that a mucin‐producing VA was initially present, from which the adenocarcinoma arose. If the presence of mucus that causes muconephrosis can be detected in the mass by ultrasonography or MRI, it may help to close in on the diagnosis of VA.

Imaging features of marked ductal dilatation due to mucin production and histopathologic and immunohistochemical findings were similar to those of low‐grade appendiceal mucinous neoplasm of the appendix and intraductal papillary mucinous neoplasm of the pancreas. GNAS and KRAS mutations frequently found in these tumors were also found in this case. No report was found on the analysis of similar genetic mutations in VA of the urinary tract, and to the best of our knowledge, this is the first report on those genetic mutations.

## 4 CONCLUSIONS

As in this case, VA of the renal pelvis can become invasive and develop into mucinous adenocarcinoma. It is critical to recognize that VA is one of the differential diagnoses of a cystic lesion with pelvic dilatation, and it has the potential for malignant transformation. The presence of irregular solid components with enhancement on CT/MRI and restricted diffusion on MRI could indicate the rare occurrence of mucinous adenocarcinoma.

## AUTHOR CONTRIBUTIONS

**Tsubasa Kawamoto:** Data curation; writing – original draft; writing – review and editing. **Masanori Ishida:** Conceptualization; investigation; resources; visualization; writing – original draft; writing – review and editing. **Takashi Yorozu:** Investigation; resources; visualization; writing – original draft; writing – review and editing. **Elly Arizono:** Writing – review and editing. **Yukari Wakabayashi:** Supervision; writing – review and editing. **Toshitaka Nagao:** Supervision; writing – review and editing. **Yoshio Ohno:** Supervision; writing – review and editing. **Kazuhiro Saito:** Supervision; writing – review and editing.

## FUNDING INFORMATION

No funding declared.

## ETHICS STATEMENT

The article describes a case report and no additional permission from our Ethics Committee was required.

## CONSENT

For the publication of this article, we obtained written informed consent from the patient to release any potentially identifiable data.

## Data Availability

The data that support the findings of this study are available from the corresponding author upon reasonable request.
